# Navigating polycythemia vera and factor X deficiency on cardiopulmonary bypass for coronary artery bypass grafting: a case report

**DOI:** 10.1051/ject/2025016

**Published:** 2025-09-15

**Authors:** Jenna L. Cornibe, Arthur H. Katz

**Affiliations:** 1 Jupiter Medical Center 1210 S Old Dixie Hwy Jupiter FL 33458 USA; 2 Perfusion Services, Epic Specialty Staffing 2250 McGregor Blvd Suite 3300 Fort Myers FL 33901 USA

**Keywords:** Bleeding disorder, Coagulation, Cardiopulmonary bypass, Cardiac surgery, CABG, Hemodilution

## Abstract

Published literature about patients with polycythemia vera (PV) undergoing cardiac surgery while utilizing cardiopulmonary bypass (CPB) is scarce, even more so when coupled with an additional rare bleeding disorder. These patients require a multidisciplinary approach to achieve optimal clinical management. Several cases have been reported of oxygenator failure or thrombosis within the extracorporeal circuit. We present a successful CPB run on a patient with coexisting PV and Factor X deficiency undergoing coronary artery bypass grafting.

## Introduction

There is a lack of published literature concerning the polycythemia vera (PV) patient population regarding cardiac surgery, particularly with the utilization of cardiopulmonary bypass (CPB). Due to both their hypercoagulable tendencies perioperatively as well as bleeding in the post-operative timeframes, these patients present a unique challenge for overall clinical management [1, 2]. Coupling PV with another existing bleeding disorder, such as Factor X deficiency, perioperative management of cardiac surgery becomes extensively complicated [3].

PV is diagnostically categorized by the Janus kinase (JAK)-2 mutation being present within a bone marrow biopsy [1]. These PV patients present with increased red blood cell (RBC) circulation from their rare myeloproliferative disease, causing possible arterial thrombosis from hyperviscosity within the blood [1]. Other common characteristics of PV include platelet (PLT) and leukocyte activation as well as fibrinolytic dysfunction, which are some of the contributing factors to this disease’s thrombogenesis [4]. Further progression of PV can lead to myeloid metaplasia or possibly leukemia [1]. Kaiser et al. previously reported a case series of PV patients who experienced oxygenator failure during their CPB run [5]. The first case ran above normal activated clotting times (ACT) and utilized hemodilution [5]. Both reported cases had either oxygenator failure and/or thrombosis seen within the CPB circuit tubing [5]. Lehot et al. also reported a PV case on CPB with above normal ACT times, which still revealed high CPB systemic arterial line pressures requiring oxygenator changeout and thrombotic CPB circuit components [6]. Furthermore, Ursulet et al. reported a patient with increasing hemoglobin (Hb)/hematocrit (HCT), which caused increasing oxygenator pressures on extracorporeal membrane oxygenation until autologous harvest and hemodilution were incorporated [7].

Factor X deficiency is also a rare blood disorder. Factor X, produced by the liver, is a vitamin K-dependent serine protease that is paramount for the formation of thrombin [8]. This deficiency can either be acquired or congenital and can be found on chromosome 13 (13q34) [8]. A patient’s Factor X level of 10–40% of normal would be deemed reasonable for hemostasis, although target levels of Factor X are not established for surgery or treatment [8]. Antifibrinolytics such as epsilon-aminocaproic acid or tranexamic acid can aid in minor bleeding cases, however, fresh frozen plasma (FFP) or alternative factor replacement concentrates would be needed for severe bleeding [9].

We report a patient with coexisting PV and Factor X deficiency receiving coronary artery bypass grafting utilizing CPB.

## Case report

A 63-year-old, 178 cm, and 87 kg male presented with coronary artery disease, PV, and Factor X deficiency noted with activity of 73% (a 1:1 mixing study revealed he should be responsive with FFP). Comorbidities included atrial fibrillation and pulmonary hypertension. He reported shortness of breath, and the patient had no known drug allergies. The patient also has a history of multiple ablations, bone marrow biopsy (JAK2 V6 17F mutation, 7q and 20q deletion), use of Jakafi^®^ (ruxolitinib) (Incyte, Wilmington, DE, USA) for which he became intolerant, and cardiomyopathy ejection fraction (EF) of 20%. Inpatient oncological and hematological workups occurred over several weeks preoperatively. The decision to place the patient on CPB versus an off-pump procedure was due to the need for a modified Cox-maze IV in addition to the coronary bypass grafts for multi-vessel disease.

A discussion was held with the entire operating team regarding the care plan for the patient. Coagadex^®^ (Coagulation Factor X, Human) (Bio Products Laboratory Limited, a Kendrion Company, Elstree, Borehamwood, UK) was preordered and held in the pharmacy. Antithrombin III (ATIII) was prophylactically brought to the operating room for standby. An entire CPB machine and disposable set were primed and ready in the sub-sterile. Pre-operative baseline labs were white blood cell count (WBC) 25.4 × 10^3^/mcL, Anti-Xa < = 0.10, and PLT 582 ×10^3^/mcL. One unit of blood, consisting of 400 mL autologous harvest, was removed prior to the chest incision. The starting in-room baseline HCT was 36.6%. The baseline heparin concentration (Hepcon) was 3.1 mg/kg utilizing the Medtronic^®^ HMS Plus Hemostasis Management System (HMS) (Medtronic, Inc., Minneapolis, MN, USA), which was increased to a prophylactic 4.0 mg/kg. The baseline ACT was 138 s. Although an ACT > 500 s was desired, achieving the Hepcon of 4.0 mg/kg was thought to be of greater value in determining adequate anticoagulation for this patient. Additionally, a decreased dose of 5.0 g of aminocaproic acid was administered in the pump prime, down from the usual 10 g dose. A femoral arterial pressure line was also placed due to compromised left ventricle function, which was the mean arterial pressure (MAP) treated and charted for this case.

A loading dose of 34,000 units (U) heparin was given, with 10,000 U added to the CPB prime. Due to a resulting 2.5 mg/kg Hepcon, while awaiting an ACT result, an additional 10,000 U of heparin was given systemically. Pre-CPB results were HCT of 37.8% and ACT of 503 s. CPB was initiated at a 2.2 L/min/m^2^ index utilizing bicaval venous cannulae and a central aortic cannula. Terumo^TM^ Advanced Perfusion System 1 with Xcoating^TM^-lined disposables (circuit, Capiox^®^ iCP Centrifugal Pump, and Capiox^®^ FX25 reservoir/oxygenator with integrated arterial filter) (Terumo Cardiovascular Systems Corporation, Ann Arbor, MI, USA) were utilized. Venous antegrade prime (VAP) was not completed with the intent to dilute blood volume. In addition, once on CPB, acute normovolemic hemodilution (ANH) with one liter of autologous blood was separated off the circuit via the quick prime line and replaced with PlasmalyteA. Arterial blood gases and ACTs were performed more frequently than standard protocol at 20-minute intervals. The next resulting ACT was 653 s with a Hepcon of 2.5 mg/kg, an additional 15,000 U of heparin was given. The HCT was 31.2%, and 300 mL of PlasmalyteA was added to decrease the HCT below 30%. Subsequent labs showed the ACT was 983 s and Hepcon was 3.5 mg/kg, an additional 15,000 U of heparin was given. HCT was 30.6%. Flow was increased to a 2.4 L/min/m^2^ index, and the patient was maintained normothermic. Standard protocol coagulation samples were drawn to be taken to the laboratory. The command was given to cool to 34 °C. Current lab values were ACT 900 s, Hepcon 3.5 mg/kg, and HCT 29.4%. An additional 5000 U of heparin were given. The aortic cross-clamp was applied. Modified Del Nido cardioplegia at a ratio of 4:1, cold 5 °C, was given with 1800 mL antegrade followed by 200 mL retrograde to achieve arrest. The previous PLT count returned at 841 × 10^3^/mcL. Repeat labs were drawn for confirmation and resulted in 828 × 10^3^/mcL. The point-of-care labs at this point revealed an ACT > 999 s, Hepcon at 2.5 mg/kg, and HCT of 27.3%. An additional 15,000 U of heparin were given due to the low Hepcon result and lack of consistent ACTs. A subsequent dose of 300 mL cold retrograde cardioplegia was delivered 30 min after the previous dose completion. The command was given to rewarm the patient to 36 °C. Current point of care results were ACT > 999 s, Hepcon at 4.5 mg/kg, and HCT at 27.6%. The aortic cross-clamp was removed.

At this point, PLT aggregation was seen around the pump suction lines. Due to this occurrence, in two separate increments, the initial separated-off pump blood volume was sent to the cell saver, washed, and returned to the pump to decrease the non-RBC components. Each occurrence yielded 500 mL washed RBCs (total of 1000 mL). A dose of 5000 U heparin was given in addition to this cell saver volume. When washing the cell saver volume, an ¼ inch white band of PLT aggregation was also seen at the top of the wash bowl. Additional saline wash volume and leukocyte reduction filters were utilized before returning to the CPB circuit. Cerebral saturation and the aortic line pressure (pump) stayed consistent throughout the entire case. The patient was uneventfully weaned from CPB with a CPB time of 154 min and cross-clamp time of 49 min; receiving a CABG ×2, modified Cox-Maze IV, and left atrial clip. Post-CPB, the patient was slowly reversed with protamine and the resulting labs were ACT 122 s, Hepcon of 0.0 mg/kg, and HCT of 32.1%. Coagulopathies were not observed while closing. The stable patient was transferred post-operatively to the cardiac intensive care unit on 0.06 mcg/kg/min of epinephrine, 40 ng/kg/min of Giapreza^®^ (angiotensin II) (Innoviva Specialty Therapeutics Inc., in partnership with La Jolla Pharmaceutical Company, Waltham, MA, USA), and 0.02 mcg/kg/min of norepinephrine. He received one unit of cryoprecipitate several hours later due to the resulting labs of Prothrombin Time (PT) 16.8 s, Partial Thromboplastin Time (PTT) 39.2 s, and INR 1.4.

The patient developed hypotension overnight. During the early morning of post-operative day (POD) 1, a stat echocardiogram was performed, revealing a hematoma and clot on the right ventricle. Lab results were WBC 72.9 × 10^3^/mcL, HCT 28%, and PLT 823 × 10^3^/mcL. Return to the operating room was required for emergent evacuation of hematoma and mediastinal exploration. Post-reoperative lab results were WBC 43.1 × 10^3^/mcL, HCT 25.7%, and PLT 557 × 10^3^/mcL. Coagadex^®^ was not administered in either operating room encounter. The patient was uneventfully extubated on POD2 and discharged POD11, on Jakafi^®^ and Eliquis^®^ (apixaban) (Bristol-Myers Squibb Company, Princeton, NJ, USA & Pfizer, Inc., New York, NY, USA).

## Discussion

Patients with PV not only present with a wide variety of coagulopathies, but coupled with the use of CPB and its hemodynamic implications, a whole subset of challenges arise. As previous literature suggests, many cases involving extracorporeal circuits have experienced issues with high CPB line pressure, oxygenator failure/dysfunction, and or thrombosis within the disposable circuit components [5–7]. Since this patient was not of an emergent nature, planning, and research took place in preparation for this case. Suggestions from an off-pump CABG case reported phlebotomizing the patient once anesthetized, as well as liberal administration of fluids during the perioperative period [10]. Our aim during the perioperative period was to stay safely anticoagulated and continue repetitive hemodilution techniques while keeping close account of his various hypercoagulable attributes. Our first step was removing a 400 mL unit of autologous blood from the patient once anesthetized to reduce RBC volume. Utilizing the HMS compared to other ACT devices, we were also able to observe the Hepcon level, which proved to be imperative in a case such as this, as the ACT itself does not appear accurate. Normally, we would run an adjusted Hepcon level at 3.0 mg/kg, but we prophylactically increased to a Hepcon of 4.0 mg/kg for this patient. This was particularly done since previously published cases with elevated CPB ACTs, showed no benefit in the prevention of thrombosis of CPB circuit components [5, 6]. Due to inconsistencies in ACT levels and their inability to produce adequate anticoagulation in previous cases [5, 6], the Hepcon level was heavily relied upon in comparison to the ACT. Heparin was liberally administered upon each Hepcon result so as not to get behind.

Standardly, before initiation of CPB, we would VAP the entire CPB circuit. It was decided to allow the hemodilution of the circuit prime in this scenario. Once CPB was initiated, we were able to separate off a liter of autologous blood, replacing it with PlasmalyteA via the quick prime line for gradual re-introduction later in the bypass run. Han et al. describe an ideal target range for HCT between 25% and 30% while on extracorporeal support [11]. However, there is also evidence an HCT below 22% of CPB can cause complications postoperatively [12]. Our goal was a target HCT < 30%, which was achieved by the addition of PlasmalyteA for ANH. Since the laboratory PLT results returned beyond the high limits with secondary confirmation as well as PLT aggregation seen in the pump suckers and the cell saver bowl, the divided-off liter of autologous blood from the beginning of the pump run was sent to the cell saver. The intent was to remove as many of the non-RBC components as possible to reduce the PLT aggregation that is starting to evolve. Extra wash volume and leukocyte reduction filters were utilized before the slow administration back to the CPB circuit with additional heparin. There was no change in pump arterial line pressure or need to increase flow that would allude to issues within the circuit/oxygenator, nor was any thrombosis seen within the enclosed part of the reservoir or CPB circuit. The cross-clamp was removed, and the patient was soon to be weaned from bypass at this point, which proved of great benefit. Cerebral saturation did not waver at any point in the perioperative period.

When the patient returned to the operating room on POD1 for re-exploration, this could have well been a bleed induced by surgical insult due to his Factor X deficiency, similarly reported in a cardiac tamponade case reported by Othman et al. [9].

In hindsight, we could have removed a second unit of autologous harvest from the patient instead of one when first anesthetized, as his HCT minimally decreased. We could have also more liberally hemodiluted with PlasmalyteA during the CPB run, aiming for an HCT in the 25% range as opposed to 30%. However, since there are scarce cases of PV reported with the use of CPB, we are hopeful that our uneventful CPB run and the way we navigated throughout may be of potential assistance for future cases of this rare blood disease.

## Data Availability

Data availability is not applicable.
